# Bilateral Lipid Keratopathy in the Setting of Brimonidine Tartrate Use

**DOI:** 10.1155/2023/8115622

**Published:** 2023-04-17

**Authors:** Majid Moshirfar, Melody Ziari, Carter J. Payne, Seth R. Stapley, Briana K. Ply, Yasmyne C. Ronquillo, Phillip C. Hoopes

**Affiliations:** ^1^Hoopes Vision Research Center, Hoopes Vision, 11820 S. State St. #200, Draper, UT, USA; ^2^John A. Moran Eye Center, University of Utah School of Medicine, 65 Mario Capecchi Dr, Salt Lake City, UT 84132, USA; ^3^Utah Lions Eye Bank, 6056 Fashion Square Dr Suite 2000, Murray, UT 84107, USA; ^4^University of Texas Health Science Center at Houston, McGovern Medical School, 6431 Fannin St, Houston, TX 77030, USA; ^5^Case Western Reserve University School of Medicine, 9501 Euclid Ave, Cleveland, OH, USA; ^6^Arizona College of Osteopathic Medicine, Midwestern University, Ocotillo Hall, 19555 59th Ave, Glendale, AZ 85308, USA

## Abstract

Lipid keratopathy (LK) is a rare disease involving lipid deposition in the cornea resulting in corneal opacification. Primary LK can arise sporadically while secondary LK is seen in patients with a history of ocular trauma, medication exposure, infection, inflammation, or disorders resulting in derangements of lipid metabolism. Secondary LK is more common and occurs due to neovascularization. Use of precipitating medications should be considered in LK workup, particularly for patients in whom other etiologies have been ruled out. Brimonidine, an ocular hypotensive medication, can be associated with LK. We present a case of bilateral secondary LK in a patient with a history of prolonged brimonidine use, without additional contributing factors.

## 1. Introduction

Lipid keratopathy (LK) refers to the deposition of lipids, including neutral fats, cholesterol, and phospholipids, into the stroma resulting in corneal opacification [1, 2]. Primary LK can occur sporadically and is seen in patients without a history of neovascularization, inflammation, or trauma. Secondary LK can occur due to a disruption in lipid metabolism, an underlying ocular disease, or a drug-related issue [2]. Secondary lipid keratopathy is more common and affects neovascularized areas of the cornea [2, 3]. Patients present with corneal opacification resulting in decreased visual acuity, in addition to features associated with the underlying causative disease [2]. We present a case of secondary lipid keratopathy in a patient with a history of long-term brimonidine tartrate (BT) use.

## 2. Case Report

An 86-year-old woman was referred by her optometrist after several months of worsening vision. Ocular history was significant for cataract surgery two years prior and a 25-year history of open-angle glaucoma with a seven-year history of treatment using brimonidine tartrate 0.1% BID OU and artificial tears (ATs). She had no history of contact lens use or eyelid disorders (i.e., styes, blepharitis, and meibomian gland disease) and no history of corneal disorders including recurrent corneal erosion, meibomian gland disease, rosacea blepharitis, blepharokeratoconjunctivitis, or ulcers. The patient reported worsening of her vision since her cataract surgery, most noticeably in the three months prior to her referral, requiring her to use a magnifying glass to read. The referring eyecare provider reported no identifiable corneal lesions prior to 2016, with slow development and progression of bilateral peripheral corneal opacities since that time. The patient also noted photosensitivity in both eyes. She had no pertinent medical history, including hyperlipidemia.

On examination, best-corrected distance visual acuity (BCDVA) was 20/60 OD and 20/70 OS. Manifest refraction was +0.75–1.00 × 160°OD and +0.50–1.25 × 165°OS, and IOP was within normal limits in both eyes. Slit lamp examination was remarkable for bilateral dermatochalasis, superficial punctate keratitis (SPK), and peripheral corneal scarring from 4 o'clock to 5 o'clock position and 7 o'clock to 8 o'clock position with superior corneal neovascularization consistent with sectoral interstitial keratitis and lipid keratopathy OU. There was no guttata or corneal lattice, but crocodile shagreen was noted in both eyes. Dilated fundus examination revealed blunted macular reflex OU and C/D ratio of 0.65 OD and 0.75 OS. Findings were consistent with bilateral lipid keratopathy secondary to interstitial keratitis. Infectious and autoimmune workup was ordered in coordination with the patient's primary care physician (PCP), including complete blood count with differential, erythrocyte sedimentation rate, C-reactive protein, auto-antibody titers (ANA, C-ANCA, and P-ANCA), rheumatoid factor, anti-SSA/Ro and anti-SSB/La, purified protein derivative (PPD) skin test and interferon-gamma release assay (IGRA) for tuberculosis, chest X-ray, ACE and lysozyme levels for sarcoidosis, throat and nasal swabs for methicillin-resistant Staphylococcus aureus, stool for ova and cysts, dental and tonsillar exam, Lyme disease titers, and syphilis testing. All tests resulted as negative or within normal limits. The patient's PCP reported no findings in the review of systems or on physical exam consistent with rheumatoid arthritis, systemic lupus erythematosus, Sjogren syndrome, or other similar systemic diseases.

**Figure 1:**
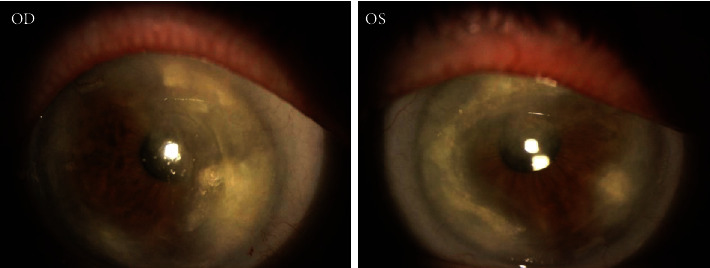
Slit lamp photographs of both eyes demonstrating significant lipid keratopathy with neovascularization of the cornea.

The patient was continued on BT and artificial tears (ATs) and then started on difluprednate drops TID OU. Slit lamp photographs taken three weeks later (shown in Figure 1) demonstrated no tangible change in the areas of lipid keratopathy. After excluding all other possibilities, we have concluded that the BT is the most likely cause of this patient's lipid keratopathy. As such, we are planning to discontinue BT in this patient; however, an appropriate alternative glaucoma treatment must be found, and we are in discussion with our glaucoma specialist to determine this.

## 3. Discussion

Lipid keratopathy (LK) is a rare disease that results in corneal opacification and vision loss [2, 3]. The exact pathophysiology is not completely understood, but there are several proposed mechanisms for secondary LK. Neovascularization results in the formation of new vessels with increased permeability; consequently, there is increased delivery of lipids to the area due to increased blood flow and increased exudation of lipids adjacent to the vessels. Lipid deposition in active LK has a characteristic fan shape [3]. Diagnosis can be made based on the patient's history, and examination findings and in vivo confocal microscopy showing deposition of cholesterol crystals can confirm the diagnosis [4]. Histochemical staining of corneal samples can confirm the presence of needle-like cholesterol crystals throughout the stroma and the deposition of neutral fats and phospholipids [4, 5]. LK must be differentiated from a corneal (or senile) arcus, an age-related finding presenting with gray opacification of the peripheral cornea due to lipid deposition. Corneal arcus, unlike LK, does not typically affect visual acuity as the central cornea is spared [1]. Bilateral corneal opacification may also be seen with Schnyder corneal dystrophy (SCD), a rare inherited disorder of the stroma. Presentation includes progressive corneal opacification with corneal arcus formation secondary to lipid deposition [2]. Consideration should be made for other diseases such as corneal xanthoma and systemic lipid disorders such as Tangier disease or familial HDL disorders, although these are less common and lack neovascularization [1–3]. Another consideration is peripheral ulcerative keratitis (PUK) which is associated with autoimmune diseases such as rheumatoid arthritis (RA) and can present with peripheral corneal stromal thinning and epithelial defects, resembling LK. We admit to the fact that a limitation of our workup was that we did not perform C3, C4, or anti-cyclic citrullinated peptide (CCP) antibody levels to better assess the potential presence of autoimmune disease. We also did not perform an anterior-segment optical coherence tomography (AS-OCT) scan to better evaluate the character and depth of the lesions, further limiting our understanding of this patient's disease process. However, in the case presented, only circumferential lipid deposition and corneal thickening were noted on exam, which are inconsistent with the peripheral thinning typically seen in RA-associated corneal disease.

Secondary LK may be drug-induced, secondary to familial lipoprotein deficiencies, or caused by either bacterial (e.g., staphylococcal marginal keratitis and styes), viral, or parasitic infections, ocular trauma (e.g., burns), or inflammation (e.g., herpetic, rosacea blepharitis, and blepharokeratoconjunctivitis). Any cause of neovascularization increases the risk of LK [2]. Various drugs have been implicated in formation of LK including brimonidine tartrate (BT), a topical ocular hypotensive agent. A summary of reported cases involving LK and BT use is shown in Table 1 [5–7]. Common ocular side effects of BT include blepharitis, allergic conjunctivitis, and conjunctival injection, while corneal complications are less common [8]. In the reported cases, patients presented with follicular conjunctivitis, conjunctival injection, neovascularization, and corneal opacities. The duration of BT therapy ranged from 18 months to 2 years, and patients were prescribed additional hypotensive medications concurrently. Allergic conjunctivitis and neovascularization reportedly improved with discontinuation of BT and initiation of steroids; however, no regression of corneal opacity was noted in any patient.

While the mechanism of LK development in patients treated with BT is unclear, it is thought to be due to an allergic reaction to some form of antigen present in BT, given the resolution with steroids, or a reaction related to preservatives such as benzalkonium and sodium chlorite found in BT [7, 8].

Other antiglaucoma topical medications besides BT have also been associated with LK, both in combination with BT and on their own. Of note, one patient was reported to have developed LK after use of latanoprost and timolol for 10 years, while another patient developed LK after taking varying combinations of seven different drops (latanoprost, tafluprost, timolol maleate, travoprost, bimatoprost, ripasudil hydrochloride hydrate, and brinzolamide/timolol maleate) over 20 years of treatment [8, 9]. Neither patient had any history of BT use.

### 3.1. Management

Management of LK focuses on addressing the underlying cause: steroid therapy to decrease inflammation and treatment of neovascularization [2]. Cessation of a potential offending medication, like BT in this case, may be warranted as appropriate, provided an alternative treatment is feasible. Reduction in neovascularization by various methods has been shown to bring about some degree of regression in lipid deposition and a modest increase in corneal clarity. These methods include subconjunctival and intracorneal bevacizumab (anti-VEGF) injections, argon laser, occlusion of neovascular vessels by fine needle diathermy (FND), and photodynamic therapy [10–13]. Recent studies have also used intravascular mitomycin-C as chemical embolization to occlude vessels and inhibit new vessel growth with subsequent partial absorption of the lipid deposits [14]. In cases where treatment was ineffective or the patient was untreated, a slow, insidious progression ensued with relatively stable visual acuity but with gradual deterioration [15]. Severe or refractory cases may require penetrating keratoplasty for definitive treatment [4].

### 3.2. Conclusion

Lipid keratopathy can be caused by a number of factors and can result in opacification and decreased vision. The patient in the case presented with corneal opacification with neovascularization in the absence of systemic disease, infection, or trauma. Consequently, her clinical picture is most consistent with LK secondary to prolonged BT use. We present this case to bring awareness to the association between LK and BT, the importance of early diagnosis due to the irreversibility of opacification, and appropriate management.

## Figures and Tables

**Table 1:** Reported cases of LK in the setting of brimonidine use.

Study	Age/gender	Eye	ToBT	Opacity	Visual acuity
Manabe et al. [5]	65/M	OS	2 y	Deep in stroma Linear, 2 to 5 o'clock	BCVA 20/20
	75/F	OS	2 y	1 to 5 o'clock Fan type	Not reported

Maruyama et al. [6]	78/F	OS	25 mo	Deep in stroma 7 to 9 o'clock Fan type	BCVA 0.15, 0.2 LogMAR after treatment
	75/F	OU	16 mo	Deep in stroma 5 to 7 o'clock OD 3 to 8 o'clock OS Fan type	Not reported

Tsujinaka et al. [7]	74/M	OU	18 mo	Deep in stroma 7 to 10 o'clock OD 3 to 6 o'clock OS	BCVA 20/100 OD and 20/80 OS

Current study	86/F	OU	7 y	4 to 5 o'clock and 7 to 8 o'clock OU	BCVA 20/60 OD and 20/70 OS

ToBT: time on brimonidine tartrate.

## Data Availability

Data sharing is not applicable to this article as no datasets were generated or analyzed during the current study.

## Additional Points

*Publication Originality Statement*. We confirm that this publication is original. *Open Access*. This article is distributed under the terms of the Creative Commons Attribution Non-Commercial 4.0 International License (http://creativecommons.org/licenses/by-nc/4.0/), which permits any noncommercial use, distribution, and reproduction in any medium, provided you give appropriate credit to the original author(s) and the source, provide a link to the Creative Commons license, and indicate if changes were made.
